# Explainable Machine Learning with Two-Layer Multi-Objective Optimization Algorithm Applied to Sealing Structure Design

**DOI:** 10.3390/ma18102307

**Published:** 2025-05-15

**Authors:** Weiru Zhou, Zonghong Xie

**Affiliations:** School of Aeronautics and Astronautics, Sun Yat-sen University, Shenzhen 518107, China; zhouwr5@mail2.sysu.edu.cn

**Keywords:** sealing structure, explainable machine learning, E-TOPSIS, FEA, IPX8

## Abstract

This study addresses the challenge of optimizing seal structure design through a novel two-stage interpretable optimization framework. Focusing on O-ring waterproof performance under hyperelastic material behavior, this study proposes a double-layer optimization method integrating explainable machine learning with hierarchical clustering algorithms. The key innovation lies in employing modified hierarchical clustering to categorize design parameters into two interpretable groups: bolt preload and groove depth. This clustering enables dimensionality reduction while maintaining the physical interpretability of critical parameters. In the first layer, systematic parameter screening and optimization are applied to the preload variable to reduce the database, with six remaining data points that constitute one-seventh of the original data. The second layer subsequently refines configurations using E-TOPSIS (Entropy Weight—Technique for Order Preference by Similarity to Ideal Solution) optimization. All evaluations are performed through FEA (finite element analysis) considering nonlinear material responses. The optimal design is a groove depth of 0.8 mm and a preload of 80 N. The experimental validation demonstrates that this method efficiently identifies optimal designs meeting IPX8 waterproof requirements, with zero leakage observed in both O-ring surfaces and motor interiors. The proposed methodology provides physically meaningful design guidelines.

## 1. Introduction

**Figure 1:**
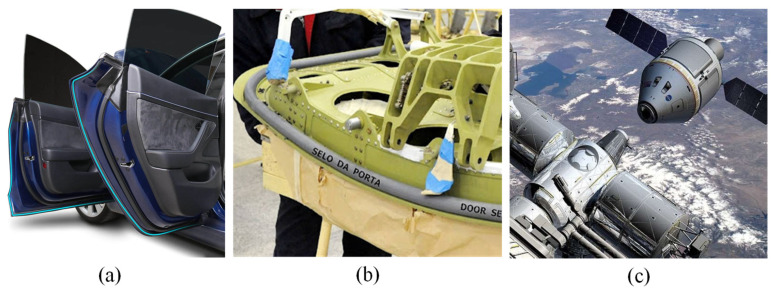
The application of sealing structures with rubber seals: (**a**) Automobile door sealing strip. (**b**) Aircraft door sealing strip. (**c**) Manned capsule docking seal structure [4].

Sealing structures with hyperelastic material are widely used in the automobile industry (Figure 1a), aviation industry (Figure 1b), and aerospace industry (Figure 1c) in sealing parts in various fields. For space stations, one of the main goals of deep-space exploration is to ensure that astronauts have a space station environment that is suitable for work and life and stops air from leaking out after docking. Sealing structures are also used in many products, such as air-pumping systems [1], the radial shaft sealing rings of production robots [2], and reciprocating sealing in marine applications [3].

The CAE method, theoretical calculation, and experimental tests are the main methods of hyperelastic material research in sealing structure design. Research in this area can be summarized through the following aspects.

In the field of composite seal design, Han Yan proposed a machine learning framework method combining finite elements and artificial intelligence for predicting the sealing and mechanical properties of fabric–rubber composites [5]. Yifeng Dong, based on the microchannel model, studied in detail the mechanical behavior and leakage characteristics of fabric–rubber composites in aircraft doors and established an explicit expression for the gas leakage rate at the contact interface of fabric–rubber composites. Through dimensional analysis, finite element analysis, and computational fluid dynamics (CFD) methods, an explicit non-dimensional function of the gas leakage rate at the contact interface of rubber seals was proposed [6]. Xiaoyao Xu studied the mechanical properties of non-orthogonal mesh fabric-reinforced rubber composites and their composite fabric rubber seals. The macro behavior of complex fabric rubber seals was studied through experiments and numerical simulations [7].

In the field of optimization of sealing performance and failure analysis, Jin Guo used the finite element software product to study the sealing characteristics of a non-standard seal and designed and optimized the structure of the non-standard seal. A new type D seal, composed of rubber and EPDM foam, was designed. The design scheme was tested and the simulation results verified the accuracy of the calculation [8]. Ganlin Cheng found that the main reason for seal failure of the ACGT-HP combined rod seal of an aircraft actuator is that various inappropriate coupling factors aggravate seal wear, resulting in a rapid reduction in contact stress and seal failure [9]. Yongjie Zhang’s research is based on experimental and numerical simulations analyzing helicopter landing pad friction faults. Three kinds of composite sealing structures were introduced to enhance the sealing performance of the buffer column, and the inherent defects of the original O-ring structure were repaired, providing an optimal contact pressure range for optimized seals [10]. Jia-Bin Wu tested the physical stress relaxation properties of nitrile butadiene rubber at a low temperature of 4 °C. The finite element numerical model of the O-ring seal structure for a deep-sea hydraulic system was established. It was concluded that the reliability of the O-ring sealing structure in a deep-sea hydraulic system continues to decline, and increasing the initial compression of the O-ring can effectively improve its sealing reliability [11]. Mei Yang et al. designed an O-ring with a skeleton seal by numerical analysis for the storage tank gate of a cutter-changing robot in large-diameter shield machines, enhancing its applicability [12]. Zhibin Zhang et al. analyzed the extrusion–occlusion dynamic failure of O-rings based on floating bushes in water hydraulic pumps [13].

In the field of mechanics behavior and contact pressure of O-rings, Yangtao Xing proposed an analytical method for investigating the contact characteristics of rubber shaft seals under dynamic eccentricity. The method improves the calculation accuracy of rubber shaft seal contact under dynamic eccentricity [14]. Zhi Chen et al. used the numerical simulation method to simulate the O-ring and concluded that excessive compression is not conducive to the sealing performance of the mechanical seal. The partial compression of the O-ring directly affects the deformation of the end face of the flexible ring [15]. Hyung-Kyu Kim used finite element analysis, experiments, and traditional theory to study the contact stress for a compressed and laterally one-side restrained O-ring [16]. Zhou et al. used the finite element analysis method and investigated the stress and contact pressure on rubber sealing O-rings under different boundary conditions [17]. Karaszkiewicz examined the geometry and contact pressure of an O-ring mounted in a seal groove [18]. Eugenio Dragoni and Antonio Strozzi provided a theoretical analysis of an unpressurized elastomeric O-ring seal inserted into a rectangular groove to describe mechanical behavior [19]. Isaac Fried and Arthur R. Johnson focused on computing the nonlinear deformation of axisymmetric solid rubber [20]. A.F. George, A. Strozzi, and J.I. Rich compared computer predictions and experimental results to investigate stress fields in a compressed unconstrained elastomeric O-ring seal [21]. Shukla and Nigam proposed a numerical–experimental analysis of the contact stress problem using full-field photoelastic data [22].

In the field of rubber sealing materials in hydrogen environments. Chilou Zhou systematically elaborated the mechanism of hydrogen-induced bubble breakage in rubber, taking into account the compression state and field performance of rubber O-rings [23,24]. Clara Clute studied five different fillers and curing agents for nitrile butadiene rubber (NBR) vulcanizates to understand the impact of additives on the performance of rubber grades suitable for high-pressure hydrogen infrastructure seals [25]. Junichiro Yamabe et al. examined the failure behavior of rubber O-rings exposed to high-pressure hydrogen gas, revealing failure modes under extreme conditions [26].

In the field of durability studies, Christopher Porter et al. critically examined the shelf life of nitrile rubber O-rings used in aerospace sealing applications and found that various physical properties of the O-ring increased with the increase in aging time [27]. Morrell P. R., M. Patel, and A. R. Skinner investigated the accelerated thermal aging of nitrile rubber O-rings, oxidation crossing leads to hardening and brittleness of the material, providing important information about material lifespan [28].

TOPSIS (Technique for Order Preference by Similarity to an Ideal Solution) has been used as a reliable tool in many fields, such as energy supplier selection [29], novel materials with anti-aging functionality [30], energy dissipation devices [31], hydrokinetic energy harvesters [32], electric vehicles [33], automotive styling design evaluation [34], multi-energy complementary heating systems [35], high-strength concrete [36], energy storage power station [37], metal inert gas welding process [38], etc. However, some studies rarely mention the application of TOPSIS algorithms in sealing structure design optimization. Finite element results under different working conditions are established to study the sealing characteristics of the O-ring sealing structure. The simulation results show different trends. Therefore, this study introduced TOPSIS to find the best design. The simulation model also provides the contour plot and database for ML and TOPSIS for optimization.

Explainable artificial intelligence (XAI) emerges as a pivotal enabler in the evolutionary trajectory from Industry 4.0 to Industry 5.0 paradigms, functioning as a foundational mechanism for demystifying algorithmic decision-making processes through enhanced interpretability, thereby mitigating epistemic uncertainties and augmenting stakeholder trust in AI-driven systems while aligning with techno ethical imperatives of transparency and accountability [39,40,41]. Explainable artificial intelligence helps engineers quickly locate the optimal geometric configuration and loading method by revealing the causal relationship between key parameters and sealing performance in the optimized design of sealing structures, significantly shortening the traditional trial-and-error cycle [42,43].

Hierarchical clustering is an unsupervised learning technique that groups data points into nested clusters using a tree-like structure (dendrogram), either merging similar clusters (agglomerative) or dividing dissimilar ones (divisive). Compared with common algorithms such as K-means and DBSCAN [44,45], it dynamically reveals multi-scale relationships without requiring prior specification of cluster count. Machine learning hierarchical clusters classify the database provided by the FEA results.

## 2. Models

### 2.1. Physical Model

**Figure 2:**
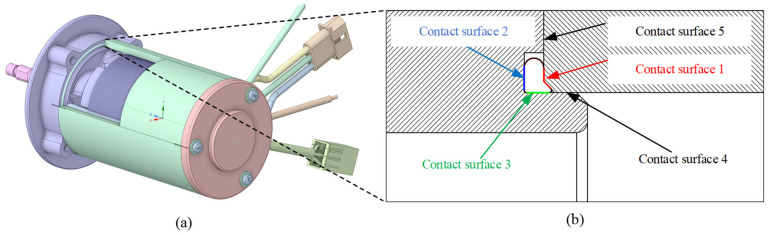
Motor model with rubber seal: (**a**) O-ring position and (**b**) contact surfaces.

Figure 2 shows a 3D motor model with the O-ring, base, and shell. There are five contact surfaces and three of them are around the O-ring. Contact surfaces 1 to 3 are the main working contact surfaces of the rubber seal. When the contact pressure of these three contact surfaces is larger than the ambient pressure (e.g., water pressure), the sealing structure works.

Another key physical quantity that is considered is the von Mises stress of the O-ring, which determines whether the rubber material works normally. If the stress is larger than the yield limit of the rubber material, then the O-ring will fail [46,47]. Formula (1) describes von Mises stress σ, where $\sigma_{1},\sigma_{2\;},\;\mathrm{and}\sigma_{3\;}$ are the principal stresses.

(1) $$\sigma =\sqrt{\frac{1}{2}[{\left(\sigma_{1}-\sigma_{2}\right)}^{2}+{\left(\sigma_{2}-\sigma_{3}\right)}^{2}+{\left(\sigma_{3}-\sigma_{1}\right)}^{2}]}$$

**Figure 3:**
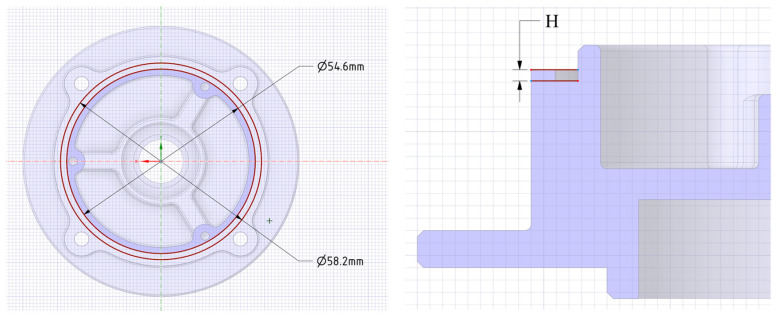
Groove dimension parameters.

The input parameters are H and preload: H is the groove depth that varies from 0.6 to 0.9 mm in Figure 3 with the diameter of the O-ring being 1.5 mm. The preload of each bolt varies from 30 N to 80 N. The FEA model is established and the mechanical performance is simulated. The FEA results include a lot of physical quantity. This paper considers the main physical quantities, which are contact area, contact pressure, and O-ring stress.

### 2.2. Numerical Model

Contact, geometric, and material nonlinearities are the main characteristics of rubber materials, and the Poisson ratio is close to 0.5 [48]. In this study, the Mooney–Rivlin model describes the hyperelastic characteristic [49,50]. The strain energy density function ψ is as follows:

(2) $$\psi =C_{10\;}\left(\bar{I_{1}}-3\right)+C_{01\;}\left(\bar{I_{2}}-3\right)+\frac{1}{d}\;{\left(J-1\right)}^{2}$$

*J* is the Jacobian, *d* is the material incompressibility parameter, and *C*_10_ and *C*_01_ are material constants; these material data are provided in the ANSYS material database, which is ANSYS Granta Selector 2023 R2 software [51,52]. $C_{10}=0.21\;MPa,\;C_{01}=0.053\;MPa,\;and\;d=0.0012\;MPa$.

In this study, the material of the motor shell is structure steel, the base and cover are aluminum alloy, and the material properties are listed in Table 1. Five contact surfaces are listed in Table 2 with the friction coefficient.

**Figure 4:**
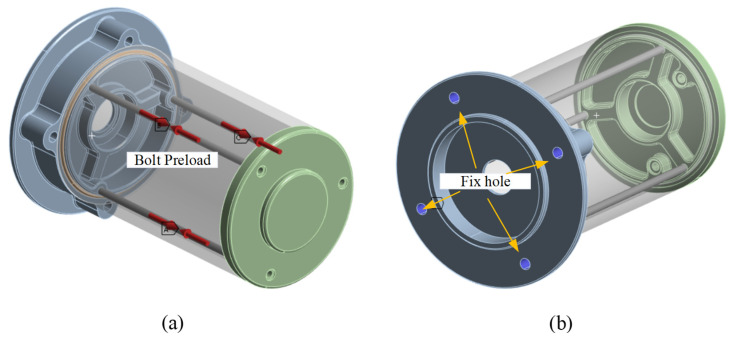
Boundary conditions: (**a**) bolt preload direction and (**b**) fix bolt hole.

Bolt preload locked the shell, base, and cover in the axial direction in Figure 4a, which makes the O-ring seal work when it compresses. The bolt hole is fixed in Figure 4b. In order to speed up the simulation and shorten the calculation time, the model used a beam connection. Beam connections ignore the geometry of bolts, washers, and nuts, which can speed up the analysis and save simulation time. In this study, the ‘Stabilization’ option and ‘Large Deflection’ option are considered.

**Figure 5:**
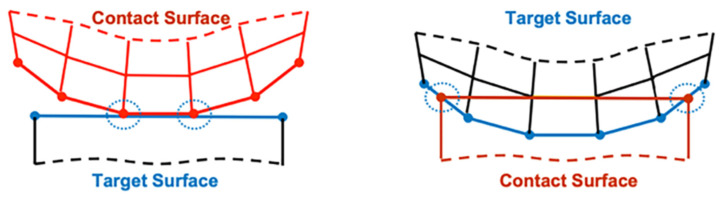
Target surface and contact surface [53].

There are 3 contact pairs around the O-ring. In the ANSYS mechanical software, each contact pairs have 2 contact surfaces which are named contact surface and target surface. The contact surface cannot penetrate the target surface. The O-ring surface is set as the contact surface, and the shell and groove surface are set as the target surfaces. The difference between the target surface and the contact surface is shown in Figure 5.

### 2.3. Grid Independence Verification

**Figure 6:**
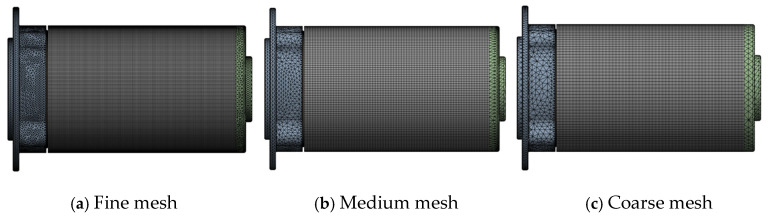
Grid independence verification.

In order to save resources, a grid independence test was carried out to find the appropriate element number while considering the accuracy and reliability of computing results and saving computing time. The coarse mesh (element number 224243), medium mesh (element number 372294), and fine mesh (element number 627654) are listed in Table 3 and Figure 6, respectively. These models consume about 1 h, 3 h, and 10 h through ANSYS workbench 2023 R2. Comparing the simulation results, the medium mesh results are close to that of the fine mesh. The fine mesh model costs more than 10 h, which can lead to the established database being time too long, and it increases the risk of computer crashes. Thus, element 372294 was selected, and the element size was applied to the other models in this research to establish a database for ML.

### 2.4. Establish Simulation Database

**Figure 7:**
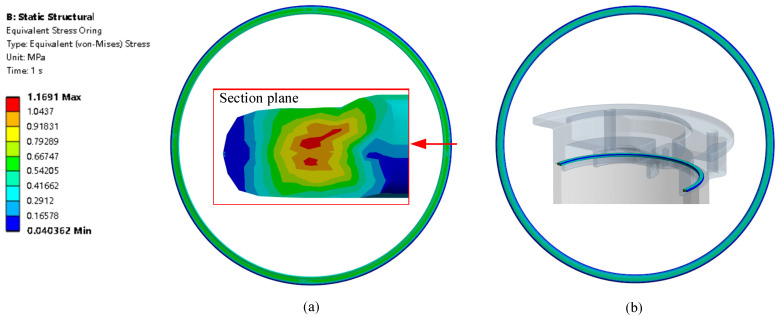
O-ring stress distribution: (**a**) O-ring CS1 side surface stress distribution and (**b**) O-ring CS2 side surface stress distribution.

There are 42 FEA models that bolt preload varies from 30 N to 80 N with the groove depth varying from 0.6 mm to 0.9 mm. The results used for evaluation are CP (contact pressure), CA (contact area), and σ (O-ring von stress) [54,55,56]. The stress distribution of the O-ring when H = 0.8 mm with bolt preload 80 N is shown in Figure 7. The maximum von Mises stress is 1.17 MPa in the center of the O-ring section, which is lower than the tensile yield stress 5.929 MPa. The section of the O-ring shows that at the chamfer location, there are some small protrusions on the inner O-ring surface.

**Figure 8:**
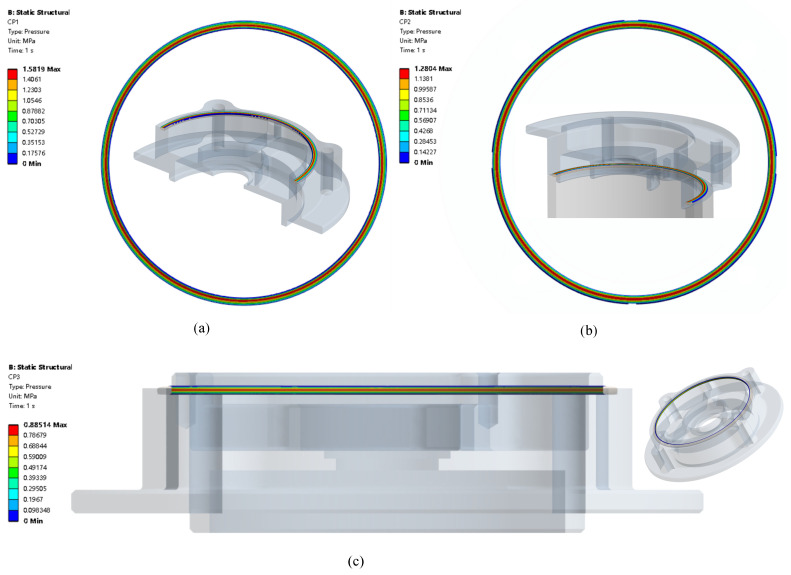
Contact pressure distribution: (**a**) CS1 contact pressure; (**b**) CS2 contact pressure; (**c**) CS3 contact pressure.

Contact pressure is very important for sealing performance. O-ring has three contact surfaces, which are shown in Figure 2b and are CS1, CS2, and CS3. The contact pressure distribution of CS1 is shown in Figure 8a, in which the max contact pressure is 1.58 Mpa, while CP2 is 1.28 MPa in Figure 8b. The max contact pressure of CS 3 is 0.89 MPa as shown in Figure 8c.

Through the above calculation, the results of the physical quantities related to the O-ring seal are obtained when the groove depth is different and the preload varies. The data are chaotic as generated by the ANSYS workbench software. In order to cluster the data, a suitable ML algorithm was introduced in this study.

### 2.5. Machine Learning Algorithms

**Figure 9:**
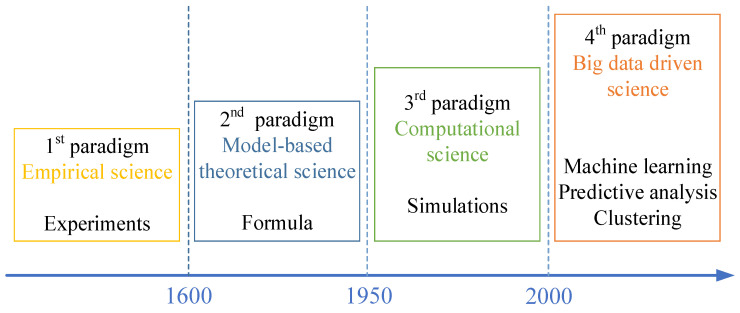
Four existing paradigms for scientific discovery.

Looking back on the history of scientific development, it is possible to distinguish four scientific discovery methodologies: (1) experiments, (2) mathematical modeling, (3) computer simulation, and (4) data analytics [57]. Figure 9 illustrates these four paradigms.

Artificial intelligence (AI) refers to a system with certain intelligence created by humans that can perform tasks that usually require human intelligence. These tasks include but are not limited to learning, reasoning, problem-solving, knowledge representation, planning, navigation, natural language processing, pattern recognition, and learning from experience. Machine learning belongs to AI which involves the use of mathematical and statistical methods to enable machines to learn from given data. These technologies include supervised, semi-supervised, unsupervised, and deep learning [58,59].

### 2.6. Data Driven by Hierarchical Clustering

Hierarchical clustering (HC) is a collection of closely related cluster algorithms that build hierarchies of clusters. Hierarchical clustering is unsupervised learning from ML which is used when there is no labeled data; it includes two basic approaches, agglomerative and divisive clustering [60]. In this paper, use the first one and it is described in Table 4 according to relevant references [61].

**Figure 10:**
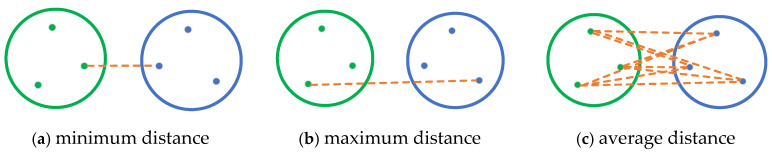
Schematic diagram of 3 cluster spacing metrics.

The core of unsupervised learning is the automatic discovery of patterns, structures, and relationships from unlabeled data. Data can be processed and analyzed without the need to label the data. At the same time, unsupervised learning can discover the inherent distribution rules and structural characteristics of data, which helps to deeply understand the nature and relationships of data. Although this method performs well in data preprocessing, anomaly detection, and dimension reduction, it also has some obvious shortcomings. For example, the results are poorly interpretable, parameter selection is difficult, calculation complexity is high, etc. Agglomerative clustering follows a bottom-up strategy in which each instance starts its own cluster. The key point is to choose the cluster merging method. There are minimum distance method, maximum distance method, and average distance method in Figure 10.

The two class clusters are defined as M and N, the data points they contain as P and P’, and the set of points they contain are denoted as C_M_ and C_N_; then, the formulas described in Figure 10 are Formulas (3)–(5), and the distance between points is calculated according to the Euclidean distance according to Formula (6) [62].

(3) $$D_{\min}\;(C_{M},\;C_{N})=\underset{P\in C_{M},P^{\prime }\in C_{N}}{\min}\left|P-P^{\prime }\right|$$

(4) $$D_{\max}\;(C_{M},\;C_{N})=\underset{P\in C_{M},P^{\prime }\in C_{N}}{\max}\left|P-P^{\prime }\right|$$

(5) $$D_{\mathrm{avg}}\;(C_{M},\;C_{N})=\frac{1}{{n_{M}n}_{N}}\;\sum_{P\in C_{M}}\sum_{P^{\prime }\in C_{N}}\left|P-P^{\prime }\right|$$

(6) $$\left|P-P^{\prime }\right|=\sqrt{\sum_{k=1}^{n}{\left(x_{k}-y_{k}\right)}^{2}}$$

**Figure 11:**
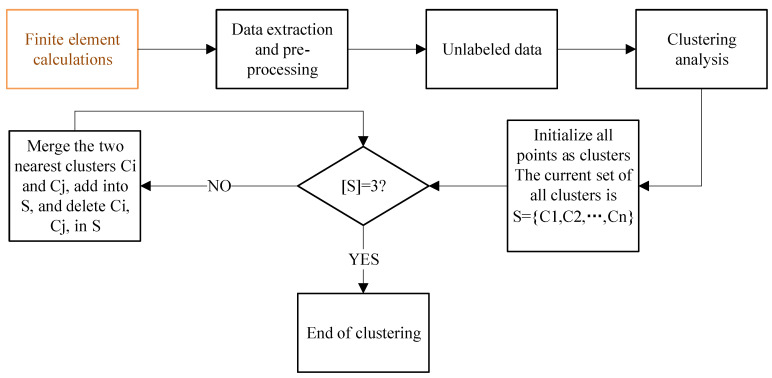
Agglomerative hierarchical clustering flow chart.

In this paper, Formula (3) calculates the distance, and S is used to represent the sample set. First, each sample is divided into a cluster, and then the distance between any two clusters is calculated, and the cluster with the smallest distance is merged until three clusters appear [63]. The flow chart in Figure 11 describes the details of how HC works on the simulation database.

**Figure 12:**
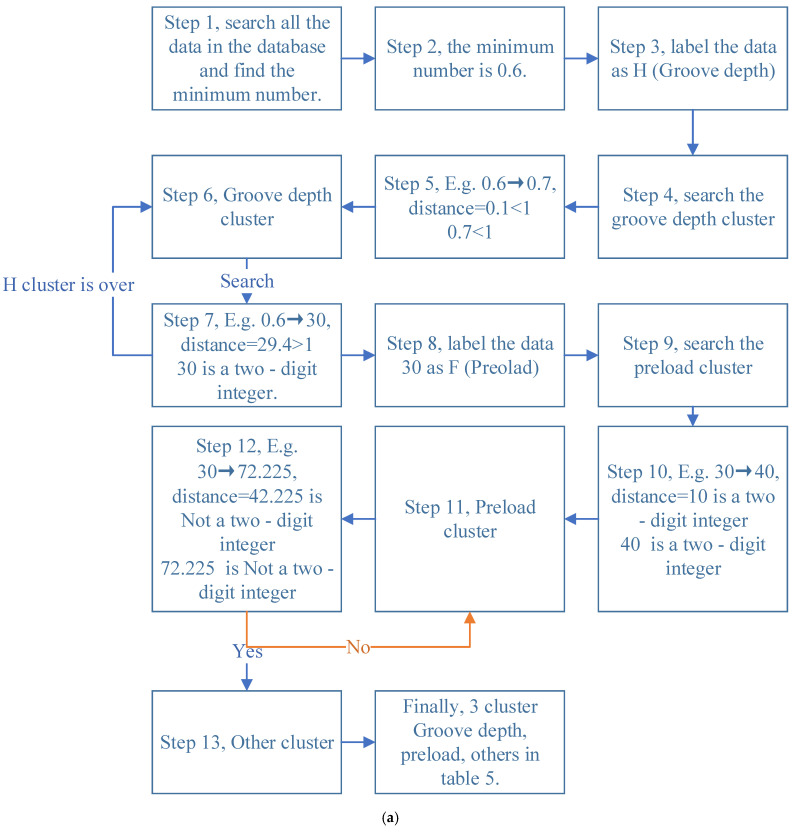

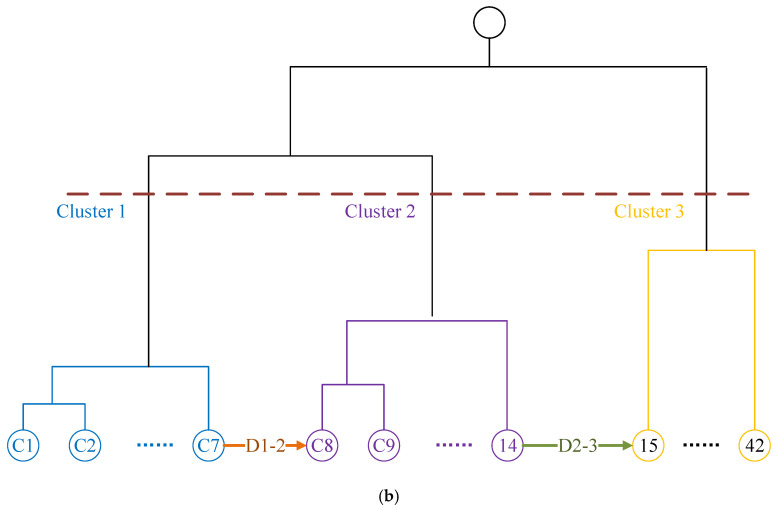
Clustering process: (**a**) schematic diagram of labeled clustering principle and (**b**) the clustering tree of hierarchical clustering.

To transform hierarchical clustering into supervised learning, after labeling the minimum data as groove depth from step 1 to step 6, the second cluster was labeled as preload from steps 7 to 11. Through these two methods, unsupervised learning transitioned to supervised learning, and an interpretable cluster was obtained. This method solves the shortcomings of unsupervised learning. At this point, the chaotic data were divided into three categories as in Figure 12: cluster 1 was groove depth (from C1 to C7), cluster 2 was preload (from C8 to C14), and cluster 3 was other data, which were the simulation results.

According to the improved hierarchical clustering algorithm of explainable machine learning, 42 finite element analysis models clustered separately, and the obtained results clustered with labels. All the results are organized in Table 5.

The data are categorized in Table 5 with the same preload with different groove depths. It is hard to choose among 42 design schemes, which is better as the results show different trends. To overcome this problem, in the next section, a multi-objective double-layer progressive optimization method is developed based on the machine learning algorithm, which is labeled the HC and TOPSIS optimization algorithm. This method considers the simulation result data of O-ring stress, the maximum contact stress, and contact area under different operating parameters.

### 2.7. Double-Layer Progressive Optimization Method

The double-layer progressive optimization method is based on the TOPSIS method as the parameters change in different trends. TOPSIS is a widely used method of multi-objective decision making to optimize parameters. The weight of each evaluation index was calculated by the entropy method.

**Figure 13:**
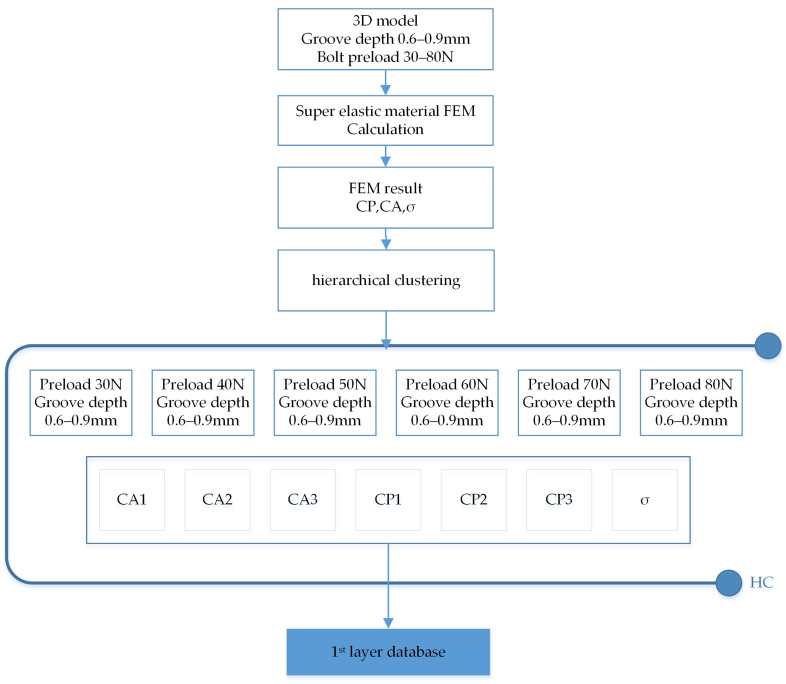
FEM calculation flow chart.

**Figure 14:**
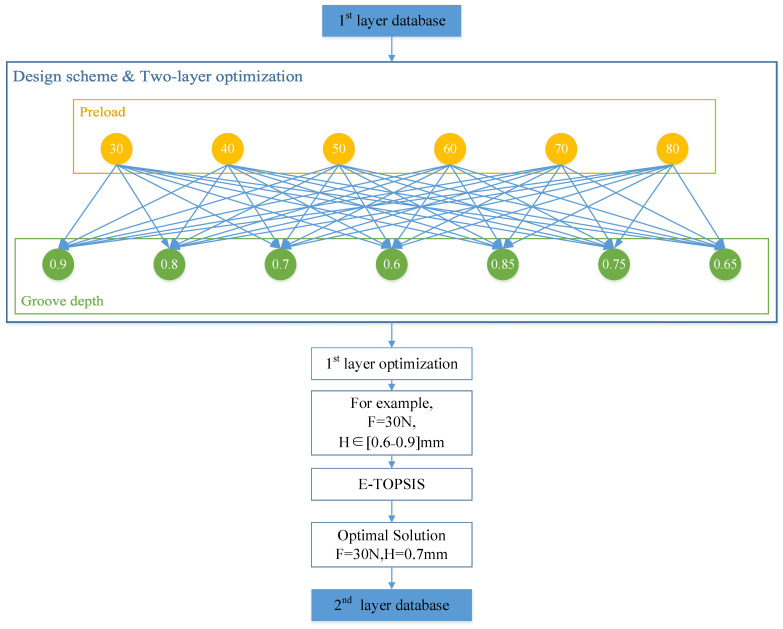
First layer database optimization flow chart.

As Figure 13 shows, the 3D model with different groove depths was built by computer 3D design software ANSYS SpaceClaim 2023 R2. Then, the model was imported into ANSYS for FEM analysis, element size was set as Figure 6b shows with the medium mesh in Table 3. The preload was started with a bolt preload of 30 Newton; an FEM model was added every 10 Newton until it stopped at 80 Newton. According to the above settings, 42 finite element analysis models were developed that consumed around 126 h. The O-ring stress (σ), O-ring contact surface pressure (CP), and O-ring contact surface area (CA) were obtained. These data make up the first layer of the dataset as Figure 13 and Figure 14’s blue squares show.

After the first layer database was ready, these data were clustered by HC, and then optimized for the first layer through the cross combination of bolt preload and groove depth. This database was transferred to the E-TOPSIS model to optimize the best design scheme for each group. In this section, the simulation result defines PIS (positive ideal solution) and NIS (negative ideal solution). According to the sealing requirements, CP and CA are PIS, and σ is NIS.

After first layer optimization, the optimal design of each preload group with different groove depths was screened out and transferred to the second layer database established in Table 6.

**Figure 15:**
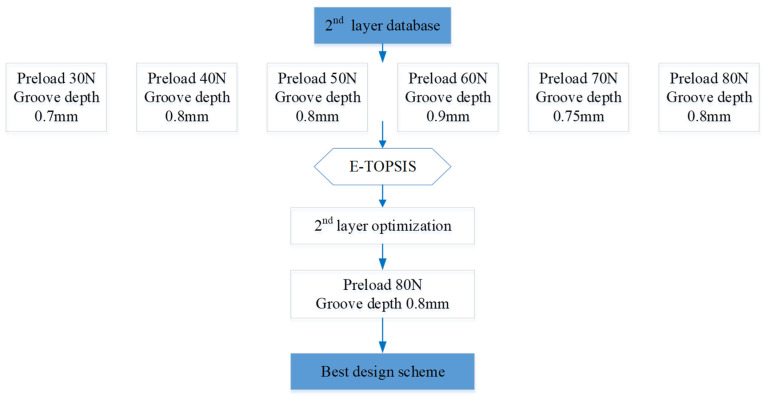
Second layer database optimization flow chart.

The basic data of the second layer optimization was formed by the E-TOPSIS method. Finally, the best design scheme was preload 80 N with a groove depth of 0.8 mm as Figure 15 shows.

#### 2.7.1. The TOPSIS Method

The TOPSIS method calculates the similarity of each data point based on the distance of the data point to the CA and CP (positive ideal solution) and the O-ring stress (negative ideal solution). The following is the workflow of this method:

Step 1: Establish the original FEA result matrix.

This matrix includes values of evaluation indices under various groove depths.

(7) $$\;\begin{array}{ccccc} C_{1} & C_{2} & C_{3} & - & C_{n} \end{array}\;\begin{array}{c} A_{1}\\ A_{2}\\ A_{3}\\ -\\ A_{m} \end{array}\left[\begin{array}{ccccc} x_{11} & x_{12} & x_{13} & - & x_{1n}\\ x_{21} & x_{22} & x_{23} & - & x_{2n}\\ x_{31} & x_{32} & x_{33} & - & x_{3n}\\ - & - & - & - & -\\ x_{m1} & x_{m2} & x_{m3} & - & x_{mn} \end{array}\right]$$

where *A_i_* (*i* = 1, 2, 3, …, *m*) is the *i*th value of H, and *C_j_* (*j* = 1, 2, 3, …, *n*) is the *j*th evaluation index (i.e., *CP*, *CA*, and *σ*).

Step 2: Normalize the FEA result matrix.

(8) $$r_{ij}=x_{ij}/\sqrt{\sum_{i=1}^{m}x_{ij}^{2}},\;i\;=\;1,\;2,\;3,\;\ldots ,\;m\;and\;j\;=\;1,\;2,\;3,\;\ldots ,\;n$$

Step 3: Through the entropy method, build the weighted normalized decision matrix.

(9) $$v_{ij}=w_{j}r_{ij},\;i\;=\;1,\;2,\;3,\;\ldots ,\;m\;and\;j\;=\;1,\;2,\;3,\;\ldots ,\;n$$

where *w_j_* is the weight for index *C_j_* in Figure 14.

Step 4: Determine the PIS and the NIS by the method below.

(10) $$PIS=\left\{\left(\underset{i}{\min}v_{ij}|j\in J_{1}\right),\left(\underset{i}{\max}v_{ij}|j\in J_{2}\right),|i=1,2,3,\ldots ,m\right\}$$

(11) $$NIS=\left\{\left(\underset{i}{\max}v_{ij}|j\in J_{1}\right),\left(\underset{i}{\min}v_{ij}|j\in J_{2}\right),|i=1,2,3,\ldots ,m\right\}$$

where *J*_1_ is the cost attributes and *J*_2_ is the benefit attributes, respectively. For example, CP is a benefit attribute that should increase.

Step 5: Compute the distances of each data to the NIS and the PIS.

Calculate the distance to the PIS by Formula (12), where $v_{j}^{+}$ is the value of the *j*th element of PIS.

(12) $$D_{i}^{+}=\sqrt{\sum_{j=1}^{n}{\left[v_{ij}-v_{j}^{+}\right]}^{2}},\;i=1,2,3,\ldots ,m$$

Calculate the distance to the NIS by Formula (13),$\;where\;v_{j}^{-}$ is the value of the *j*th element of NIS.

(13) $$D_{i}^{-}=\sqrt{\sum_{j=1}^{n}{\left[v_{ij}-v_{j}^{-}\right]}^{2}},\;i=1,2,3,\ldots ,m$$

Step 6: Obtain the similarity of each alternative to the ideal solution.

(14) $$C_{i}^{*}=\frac{D_{i}^{-}}{D_{i}^{+}+D_{i}^{-}},\;i=1,2,3,\ldots ,m$$

In Formula (14), C_i_^*^ ∈ [0, 1].

Step 7: Select the design scheme with $C_{i}^{*}$ closest to 1 as optimal design.

#### 2.7.2. The Entropy Method

The entropy method calculates the weights of indices according to the amount of information. It provides the weights for TOPSIS including five steps:

Step 1: Normalize the rating matrix, that is, the simulation results. It includes CP, CA, and σ.

For benefit indices (CP3, CP2, CP1, CA3, CA2, and CA1):

(15) $${x_{ij}}^{\prime }=\frac{x_{ij}-x_{j}^{min}}{x_{j}^{max}-x_{j}^{min}},i=1,2,3,\ldots ,m$$

For cost indices (σ):

(16) $${x_{ij}}^{\prime }=\frac{x_{j}^{max}-x_{ij}}{x_{j}^{max}-x_{j}^{min}},i=1,2,3,\ldots ,m$$

Step 2: Calculate the contribution of the *i*th alternative in the *j*th index

(17) $$p_{ij}={x_{ij}}^{\prime }/\sum_{i=1}^{m}{x_{ij}}^{\prime },\;i\;=\;1,\;2,\;3,\;\ldots ,\;m\;and\;j\;=\;1,\;2,\;3,\;\ldots ,\;n$$

Step 3: Compute entropy for each index, *E_j_* ∈ [0, 1]

(18) $$E_{j}=-K\sum_{i=1}^{m}p_{ij}\ln p_{ij},\;K=1/\ln m,j=1,2,3,\ldots ,n$$

Step 4: Compute $d_{j}$ (degree of divergence) according to *E_j_*

(19) $$d_{j}=1-E_{j}$$

Step 5: Compute the weight of CP, CA, and σ

(20) $$w_{j}=d_{j}/\sum_{k=1}^{n}d_{k}$$

## 3. Results and Discussion

The simulation time is 126 h following a computational analysis using ANSYS Workbench 2023 R2 on a workstation equipped with an Intel^®^ Xeon^®^ W-2275 processor (3.30 GHz) and 256 GB RAM. The generated simulation data points are clustered by running relevant Python code in PyCharm 2024.2.1 (Community Edition). The data generated by the finite element method are random and disorderly, which makes finding correlations in these data and obtaining optimal designs under the given conditions difficult.

### 3.1. Calculate the Best Design Solution

After optimizing the first layer of data, the optimization results are transferred to the next layer for secondary optimization so as to find the best design solution among the 42 designs.

Table 6 shows the second layer database for optimization with varying parameters, which are CP, CA, and σ, under various groove depths and preloads in order to find the best design of these six designs. Therefore, the second layer of data is optimized again with TOPSIS.

**Figure 16:**
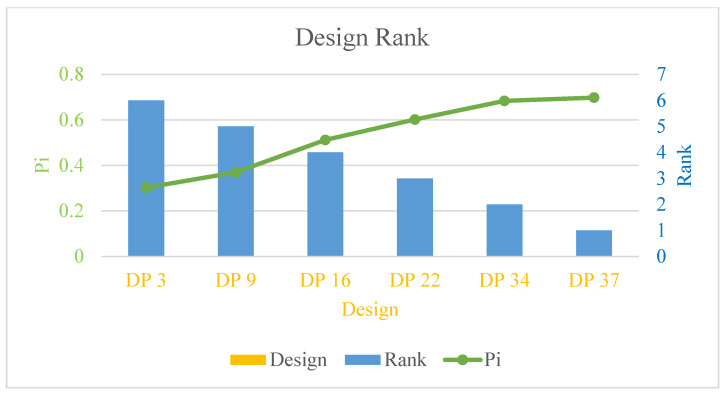
Similarities and rankings of different designs.

The similarities and corresponding ranks of the second database are listed in Table 7. The optimal design is DP 37 Pi is the largest among all the designs. The similarities of DP37 and DP34 are very close to each other. Figure 16 shows the relationship between these parameters.

### 3.2. Experimental Verification

According to Figure 16, the optimal design scheme is DP37. Among all the optimal design schemes, the optimal groove depth of 0.8 mm appeared three times, which is the highest in frequency. By comparing the three design schemes of DP37, DP16, and DP9, under the premise of the same groove depth, the greater the bolt preload, the higher the ranking. Based on the above, a prototype was manufactured according to the parameter of DP37 to test the waterproof sealing performance.

Based on the data of DP37 in Table 6, CP3 = 890.458 kPa; CP2 = 1285.460 kPa; CP1 = 1575.152 kPa. The contact pressures all exceed a water pressure of 15 kPa. The simulation results indicate that the waterproof requirement can be met.

In order to test the sealing structure reliability of the combination optimization method, this study adopts a waterproof sealing test, which has the advantages of low price and cost, and easy implementation. According to IEC 60529 and ISO 20653:2013, IPX8 is more severe than IPX7, defined as a waterproof test under 1.5 m water for 1 h [64].

**Figure 17:**
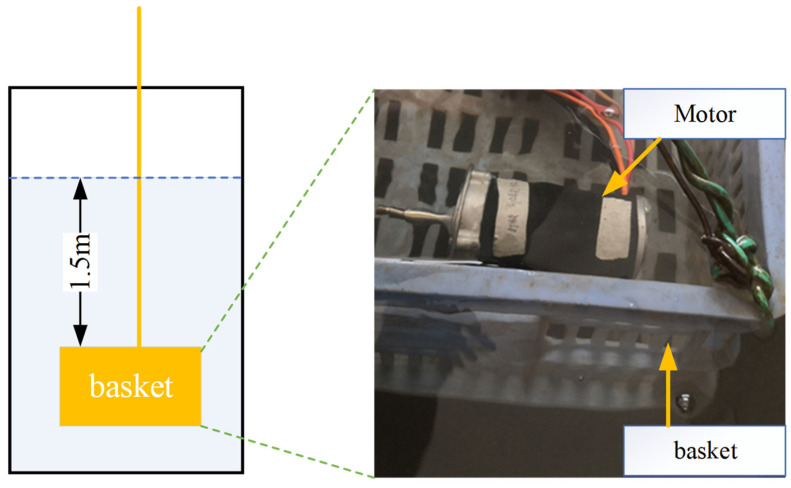
IPX8 waterproof test.

The waterproof test was carried out by manufacturing a motor prototype with the sealing structure parameters in DP 37 and putting it into a basket under 1.5 m water in a water tank as Figure 17 shows. All the gaps were sealed with sealant except for the O-ring seal construction.

Gravity, which causes the fluid pressure in a closed container, has the relationship of P (water pressure) with the following formula:

(21) P=ρgh

ρ is the density of water taken as $\rho =1000\;kg/m^{3}$, g is the acceleration of gravity taken as g = 10 m/s^2^, and h is the depth of immersion water taken as h = 1.5 m.

From the theoretical calculation, when the basket is immersed underwater at 1.5 m, the water pressure is 15 kPa. The basket with the test motor was submerged 1.5 m under water and kept for 1 h. Then, the motor was picked up, and the water stains on the surface of the motor were wiped off. The motor was put on the test bench for disassembly.

**Figure 18:**
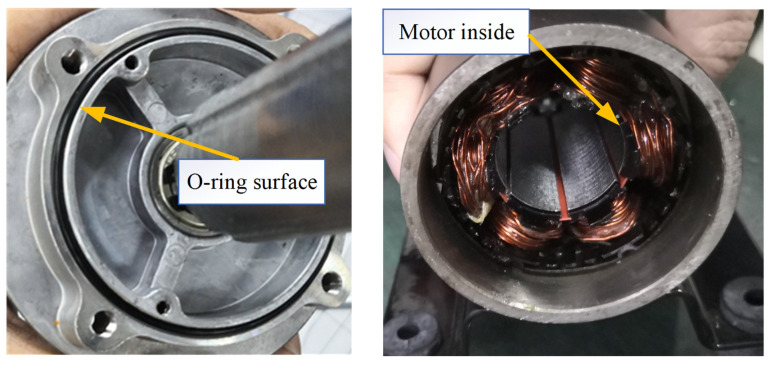
Check the waterproof performance.

As Figure 18 shows, the rubber surface is clean without water stains, and the motor base is partially clean without water droplets. The inside of the motor is dry, and there are no water droplets or water stains.

## 4. Conclusions

In this paper, by marking the minimum value as H, the transformation of the hierarchical clustering of unsupervised learning into an interpretable supervised learning model was carried out. Through this method, the data generated by the finite element method was clustered and turned from disorderly data into clear hierarchical data. Then, the database of each layer was optimized layer by layer, and finally, the best design solution (DP37) among all 42 designs was found. Artificial intelligence algorithms and optimization methods were combined to find optimal results, and the waterproof reliability of the DP37 was verified through the IPX8 waterproof test. The conclusions are summarized as follows:

1. This paper improved hierarchical clustering in machine learning and transformed unsupervised learning into supervised learning by labeling the minimum value as the groove depth, storing the distance, and comparing the distance between clusters. The categories of clusters were divided into three categories (groove depth, bolt preload, and other). At the same time, it solved the unexplainably of hierarchical clustering, making the clustered data interpretable. This lays a reliable data foundation for the next step of optimization.
2. By introducing the E-TOPSIS method, two layers of data were used for progressive optimization. After two-stage progressive optimization, the best design solution was found to be DP37. The optimization results indicate that the minimum groove depth must be at least 0.7 mm. The groove depth of 0.8 mm appeared three times, which can be given priority consideration during the design process. Under different preloads, the corresponding optimal designed groove depths are different.
3. The O-ring contact pressure far exceeded the waterproof level requirements of IPX8. However, the stress on the O-ring part was also high at this time. Based on the data of DP37 in Table 6, CP3 = 890.458 kPa; CP2 = 1285.460 kPa; CP1 = 1575.152 kPa. The contact pressures all exceeded the water pressure of 15 kPa.
4. By manufacturing a motor prototype with the sealing structure, the most stringent IPX8 waterproof test was carried out to test the water resistance performance and durability of the sealing structure. The experimental results verified that the waterproof structure is reliable.

The method proposed in this paper enables the rapid and precise selection of optimal design schemes while ensuring clarity and interpretability throughout the entire process. By integrating the finite element method (FEM), the computational efficiency was significantly enhanced, reducing the time, workforce, and material resources traditionally required for experimental validation. Furthermore, in this study, by introducing explainable artificial intelligence (XAI) technology, the limitation of hierarchical clustering methods that produce difficult-to-explain results was addressed. This integration not only improves transparency but also strengthens the reliability of decision making in sealing structural optimization. The method proposed in this paper can easily be applied in motor sealing structure design and extended to other fields such as sealing in aerospace and marine applications.

## Figures and Tables

**Table 1:** Material properties.

	E (MPa)	*μ*	R_p0.2_ (MPa)	*R_m_* (MPa)
Aluminum Alloy	71,000	0.33	280	310
Structure Steel	200,000	0.3	899	1029

**Table 2:** Friction coefficient of different contact surfaces.

Contact surface	1	2	3	4	5
Friction coefficient	0.18	0.18	0.18	0	0.15

**Table 3:** Grid independence test.

	Coarse	Medium	Fine
Mesh Elements	224,243	372,294	627,654
Stress O-ring [MPa]	1.176	1.172	1.167
CA3 [mm^2^]	149.829	149.392	149.068
CA2 [mm^2^]	225.143	224.698	224.924
CA1 [mm^2^]	191.447	193.008	192.417
CP3 [MPa]	0.888	0.890	0.991
CP2 [MPa]	1.281	1.285	1.285
CP1 [MPa]	1.708	1.575	1.562
Time	45 m 55 s	3 h 2 m 24 s	10 h 44 m 38 s

**Table 4:** Agglomerative hierarchical clustering algorithm.

Algorithm
1. Calculate the proximity matric
2. repeat
3. Merge the two closest clusters
4. Calculate the proximities of the new clusters and update the proximity matrix
5. until only one cluster remains

**Table 5:** Operating parameters and simulation results.

Name	H (mm)	F (N)	CP3 [kPa]	CP2 [kPa]	CP1 [kPa]	CA3 [mm^2^]	CA2 [mm^2^]	CA1 [mm^2^]	σ [kPa]
DP 1	0.9	30	373.260	675.264	826.319	72.006	167.804	154.944	574.913
DP 2	0.8	30	369.214	688.199	826.690	72.225	166.941	157.407	576.109
DP 3	0.7	30	370.842	687.331	816.346	72.075	169.550	160.428	575.485
DP 4	0.6	30	371.457	669.050	841.629	72.041	171.618	158.965	575.223
DP 5	0.85	30	370.961	690.962	823.564	72.061	166.246	159.543	575.679
DP 6	0.75	30	369.993	686.830	830.894	72.224	169.682	157.589	575.509
DP 7	0.65	30	369.907	692.456	824.416	72.061	166.372	159.585	575.531
DP 8	0.9	40	501.603	798.044	960.637	79.134	210.220	179.782	690.961
DP 9	0.8	40	499.042	798.469	962.185	76.232	211.258	180.383	689.828
DP 10	0.7	40	501.209	801.164	957.897	78.580	210.034	179.792	690.855
DP 11	0.6	40	505.202	795.156	960.193	77.267	210.354	179.671	691.086
DP 12	0.85	40	501.934	799.923	959.311	77.928	210.295	179.985	690.920
DP 13	0.75	40	500.742	797.821	962.383	75.397	211.107	180.341	690.058
DP 14	0.65	40	500.719	794.698	968.123	79.108	210.121	180.103	690.554
DP 15	0.9	50	639.394	920.830	1091.699	112.266	217.229	183.018	815.337
DP 16	0.8	50	624.581	919.214	1097.374	112.680	216.762	183.413	814.861
DP 17	0.7	50	619.620	879.550	1097.117	112.380	216.861	183.196	815.676
DP 18	0.6	50	609.178	919.706	1097.101	112.306	217.098	182.992	815.565
DP 19	0.85	50	603.734	919.518	1084.724	112.282	217.202	182.901	815.847
DP 20	0.75	50	604.613	919.744	1092.095	112.751	216.681	183.551	814.143
DP 21	0.65	50	623.532	916.317	1097.975	112.320	217.121	182.981	814.348
DP 22	0.9	60	698.192	1046.144	1238.917	122.735	219.860	186.264	935.005
DP 23	0.8	60	702.336	1037.805	1268.910	122.254	219.032	187.859	935.901
DP 24	0.7	60	726.270	1037.327	1231.650	121.310	219.670	186.285	934.995
DP 25	0.6	60	708.632	1036.759	1208.272	122.898	219.791	186.046	936.117
DP 26	0.85	60	703.503	1042.294	1211.723	124.981	220.055	185.485	935.148
DP 27	0.75	60	699.472	1041.834	1216.214	123.183	219.311	186.823	934.185
DP 28	0.65	60	704.650	1036.944	1235.403	123.786	219.483	185.894	940.533
DP 29	0.9	70	800.875	1167.965	1401.802	138.469	222.490	189.135	1052.715
DP 30	0.8	70	790.563	1141.248	1380.084	140.672	221.716	190.093	1052.153
DP 31	0.7	70	818.199	1161.897	1404.293	136.603	222.665	188.991	1052.687
DP 32	0.6	70	795.455	1164.512	1420.487	137.813	222.546	189.148	1052.495
DP 33	0.85	70	805.635	1174.407	1429.757	129.231	222.470	189.776	1051.409
DP 34	0.75	70	792.773	1160.420	1409.919	138.118	222.268	189.840	1050.239
DP 35	0.65	70	794.498	1169.693	1422.378	141.213	222.794	188.399	1052.527
DP 36	0.9	80	890.485	1300.138	1603.652	146.723	225.751	191.408	1170.292
DP 37	0.8	80	890.458	1285.460	1575.152	149.392	224.698	193.008	1171.645
DP 38	0.7	80	886.455	1273.428	1570.733	138.186	225.510	191.295	1170.762
DP 39	0.6	80	879.779	1285.880	1595.158	149.193	225.728	191.310	1168.325
DP 40	0.85	80	916.426	1290.929	1551.704	148.123	225.403	191.399	1179.045
DP 41	0.75	80	887.952	1274.425	1578.789	143.130	225.208	191.787	1167.400
DP 42	0.65	80	887.330	1280.772	1577.947	149.350	225.548	191.336	1169.491

**Table 6:** Second layer database.

Name	H (mm)	F (N)	CP3 [kPa]	CP2 [kPa]	CP1 [kPa]	CA3 [mm^2^]	CA2 [mm^2^]	CA1 [mm^2^]	σ [kPa]
DP 3	0.7	30	370.842	687.331	816.346	72.075	169.550	160.428	575.485
DP 9	0.8	40	499.042	798.469	962.185	76.232	211.258	180.383	689.828
DP 16	0.8	50	624.581	919.214	1097.374	112.680	216.762	183.413	814.861
DP 22	0.9	60	698.192	1046.144	1238.917	122.735	219.860	186.264	935.005
DP 34	0.75	70	792.773	1160.420	1409.919	138.118	222.268	189.840	1050.239
DP 37	0.8	80	890.458	1285.460	1575.152	149.392	224.698	193.008	1171.645

**Table 7:** Similarities and rankings of different designs.

Design	DP 3	DP 9	DP 16	DP 22	DP 34	DP 37
Pi	0.302	0.370	0.512	0.601	0.683	0.698
Rank	6	5	4	3	2	1

## Data Availability

The original contributions presented in this study are included in the article. Further inquiries can be directed to the corresponding author.

## Nomenclature

**Symbol**	**Unit**	**Implication**
*CP*	[kPa]	contact pressure
*CP1*	[kPa]	contact pressure of Contact surface 1
*CP2*	[kPa]	contact pressure of Contact surface 2
*CP3*	[kPa]	contact pressure of Contact surface 3
*CA*	[mm^2^]	contact area
*CA1*	[mm^2^]	contact area of Contact surface 1
*CA2*	[mm^2^]	contact area of Contact surface 2
*CA3*	[mm^2^]	contact area of Contact surface 3
*σ*	[MPa]	von Mises stress
σ_1_	[MPa]	principal stresses at x direction
σ_2_	[MPa]	principal stresses at y direction
σ_3_	[MPa]	principal stresses at z direction
*E*	[MPa]	Young’s modulus
*μ*	[-]	Poisson’s Ratio
*R_p0.2_*	[MPa]	Tensile Yield Strength
*R_m_*	[MPa]	Tensile Ultimate Strength
*CS1*	[-]	Contact surface 1
*CS2*	[-]	Contact surface 2
*CS3*	[-]	Contact surface 3
*CS4*	[-]	Contact surface 4
*CS5*	[-]	Contact surface 5
*μk*	[-]	kinetic frictional coefficient
*P*	[kPa]	water pressure
g	[m/s^2^]	Earth’s gravity
h	[m]	submersion in water depths
*H*	[mm]	groove depth
*x*, *y*, *z*	[m]	Cartesian coordinates
*COF*	[-]	coefficient of friction
*HC*	[-]	hierarchical clustering
*ML*	[-]	machine learning
*XAI*	[-]	explainable artificial intelligence
*NBR*	[-]	nitrile butadiene rubber
*ρ*	[kg/m^3^]	water density
FEM	[-]	finite element method
IP	[-]	Ingress Protection
NIS	[-]	negative ideal solution
PIS	[-]	positive ideal solution
TOPSIS	[-]	Technique for Order Preference by Similarity to an Ideal Solution similarity to ideal solution
